# The Neural Correlates of Consciousness and Attention: Two Sister Processes of the Brain

**DOI:** 10.3389/fnins.2019.01169

**Published:** 2019-10-31

**Authors:** Andrea Nani, Jordi Manuello, Lorenzo Mancuso, Donato Liloia, Tommaso Costa, Franco Cauda

**Affiliations:** ^1^Focus Lab, Department of Psychology, University of Turin, Turin, Italy; ^2^GCS-FMRI, Koelliker Hospital and Department of Psychology, University of Turin, Turin, Italy; ^3^Neuroscience Institute of Turin, University of Turin, Turin, Italy

**Keywords:** consciousness, attention, neural correlates, fronto-parietal network, global workspace, brain network, synchronization

## Abstract

During the last three decades our understanding of the brain processes underlying consciousness and attention has significantly improved, mainly because of the advances in functional neuroimaging techniques. Still, caution is needed for the correct interpretation of these empirical findings, as both research and theoretical proposals are hampered by a number of conceptual difficulties. We review some of the most significant theoretical issues concerning the concepts of consciousness and attention in the neuroscientific literature, and put forward the implications of these reflections for a coherent model of the neural correlates of these brain functions. Even though consciousness and attention have an overlapping pattern of neural activity, they should be considered as essentially separate brain processes. The contents of phenomenal consciousness are supposed to be associated with the activity of multiple synchronized networks in the temporo-parietal-occipital areas. Only subsequently, attention, supported by fronto-parietal networks, enters the process of consciousness to provide focal awareness of specific features of reality.

## Introduction

During the last three decades the advent and development of new scientific procedures, such as the functional magnetic resonance imaging (fMRI) and the positron emission tomography (PET), have allowed neuroscientists to study the activity of the living brain. These methods have been extensively used to determine the activation of brain regions in connection with specific cognitive functions. Although these tools of scientific investigation have significant limitations in terms of temporal resolution (Raichle, 1998; Logothetis and Wandell, 2004), they nonetheless have good performances in spatial resolution. Research programs across all over the world have used them to identify with an acceptable degree of accuracy the neural correlates of any aspect of mental activity. The outcome of this massive endeavor relies not only on the technological power of these instruments, but also on our correct interpretation of the neuroimaging results. As a matter of fact, a correct analysis of experimental data is of fundamental importance, particularly when human cognitive functions are studied.

Neural correlates can be described at very different scales, depending on the applied method. Electrophysiological techniques allow to record signal even from a single cell, and neuronal arrays can track real-time interactions at the same micro-scale. Conversely, neuroimaging instruments are useful to study phenomena from meso- to macro-scale. The MRI research constantly tries to improve spatial resolution by reducing the size of voxels; however, spatial resolution still remains very far from cellular details. The fMRI methods allow to investigate brain functions under two conditions: resting state and task. During the resting state we can analyze the spontaneous activity (which is sometimes referred to as “intrinsic activity”) of the brain while the subject is not engaged in a specific task (Biswal, 2012). During the task condition (which is sometimes referred to as “extrinsic activity”) we can analyze the state of an individual’s brain while he or she is engaged in an experimental paradigm, which often involves the presentation of sensory stimuli (Clark, 2012) – for a detailed comparison of the two conditions see Smitha et al. (2017). Both resting state and task experiments are frequently based on the measurement of the so-called blood oxygenation level dependent (BOLD) effect (Ogawa et al., 1992). The BOLD signal represents the changes in the level of oxygenation of a brain region related to neuronal activations (Pike, 2012). Consequently, it is supposed to roughly reflect the underlying electrical activity, though neurovascular coupling is still an open topic of debate (Pike, 2012). As the BOLD signal is a quasi-quantitative and indirect measurement of these activations (Pike, 2012; Robertson and Williams, 2016), fMRI does not allow *stricto sensu* the direct manipulation of neuronal firings. However, in task conditions it is assumed that changes of the BOLD signal shortly before the presentation of a stimulus correlate with brain activity related to the processing of that stimulus. Hence, fMRI may to some extent allow the detection of the effect due to an ongoing manipulation.

A further and more stringent question pertains to the specificity of the interpretation of a pattern of brain activity in terms of mental functions. In other words, how confident can we be when we assign to an observed activation pattern a specific function? This is often called “reverse inference” and a dissertation of this topic is beyond the scope of this review (for an in-depth analysis see Cauda et al., 2019). The debate about the possibilities, as well as difficulties, of reverse inference is currently ongoing, involving not only neuroscientific aspects, but also philosophical and mathematical ones. However, this method should be applied with caution when dealing with neuroimaging data (Poldrack, 2006).

In theory, all mental activities might be mapped on the brain and associated with a specific neural correlate (Nani et al., 2013). This neural stance (Lamme, 2006) is supposed to be at the basis of neuroscientific research: a change in the mind (here broadly conceived as the collection of all the intellectual processes capable of producing behavioral manifestations, thoughts, and feelings) must be always accompanied by a change in the brain. However, if this is now beyond dispute, it is not the other way round: a change in the brain may not always be accompanied by a change in the mind. In other words, the relationship between mind and brain appears to be not symmetrical. From a macro-scale perspective, different mental functions are supposed to be associated with different neural correlates; however, different neural correlates might be associated with the same mental function (for instance, pain can be processed and felt differently from person to person, or even by the same person in different times). Furthermore, there is the tricky question to conceptually distinguish a mental function from the others. Are two mental functions really distinct or one can subsume the other? Ideally, we should be able to define them clearly, which happens very rarely (memory, for instance, has been successfully divided into different types – short-term, long-term, episodic, semantic, procedural, etc. –, but other mental functions, such as language, are so multifaceted and complex that defy any attempt of precise classification). In light of these theoretical quandaries, it is still controversial whether specific neural correlates can be matched precisely to each of the numerous and various aspects of the human mind.

We can see how theoretical and empirical issues are thickly intertwined in the quest for understanding consciousness and attention (Koch, 2006). Although research has been extensive on both sides, so far there is no agreement on their definition. Some authors consider attention the sentry at the gate of consciousness (Zeman, 2001), a fundamental prerequisite for being conscious of something (James, 1890; Posner, 1994; Velmans, 2000; O’Regan and Noe, 2001). According to this view, attention should be thought of as a type of focal awareness and, as a consequence, the concept of attention should be absorbed in the concept of consciousness. In contrast, other researchers have provided evidence that consciousness and attention might be distinct and separate processes going on in the brain (Baars, 1997; Damasio, 1999; Koch and Tsuchiya, 2007; Dehaene and Changeux, 2011). According to this view, although we need attentional selection to get a full conscious access of stimuli, attention and consciousness should be treated as dissociable brain processes and, therefore, we should maintain their distinction at the conceptual level.

**FIGURE 1:**
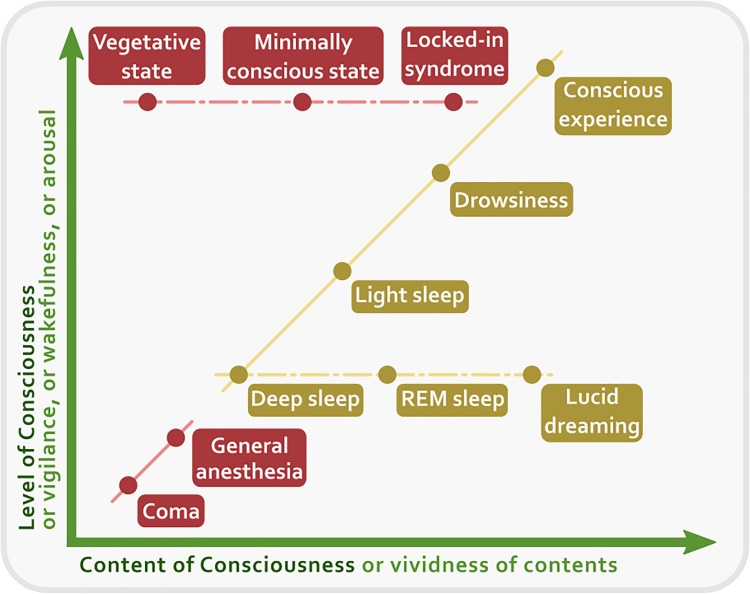
A two-dimensional representation of consciousness. The *X* axis represents the Content of Consciousness, the experience of which can vary in vividness. The *Y* axis represents the Level of Consciousness, or wakefulness, or vigilance, or arousal. Clinical conditions are in red, while normal physiological states are in yellow. Solid lines indicate transitions between states that require a change both of content and of level of consciousness. Conversely, dashed lines indicate transitions for which one dimension remains constant.

At least from the psychological point of view, it could be argued that consciousness and attention seem to refer to different concepts and mental activities. Consciousness is a broader label than attention, so much so that we can roughly single out at least three general conceptions of consciousness: consciousness as ‘waking state,’ consciousness as ‘experience,’ and consciousness as ‘mind’ (Zeman, 2001). But other distinctions are possible. For instance, given its different contents, we can distinguish a ‘phenomenal consciousness’ and an ‘access consciousness’ (Block, 1995). The former is related to how reality appears to us, while the latter is related to the cognitive availability of certain information. In this review we will adopt this distinction and the framework of the two dimensions of consciousness, that is, the dimension of wakefulness and the dimension of contents of conscious experience (Cavanna et al., 2013). We consider phenomenal consciousness as the way the world appears to us, that is, as the collection of all the possible qualitative features of reality. In turn, we consider access consciousness as the availability of a specific content of consciousness, of which we can become focally aware. These two aspects of consciousness (phenomenal and focal) conflate with each other under the control of attention and can find their place in a Cartesian diagram at the intersection of two points, one related to the level of wakefulness, vigilance, or alertness, and another related to the level of intensity, vividness, and focality of each phenomenal content. In theory, every degree of conscious experience can be represented in this two-dimensional space (Figure 1).

The concept of attention is no less clear-cut, but can be analytically sifted as much as the concept of consciousness. Attention can be selective, that is, it can be focused on a particular stimulus or object of the world. It can be exogenous when it is elicited by an external stimulus, or endogenous when it is elicited by an internal one. It can be involuntary if it is captivated by an abrupt and sudden stimulus, or it can be voluntary if it is intentionally concentrated on a certain thing. Attention can last very briefly, passing from stimulus to stimulus, or can be sustained for a long time toward a specific object. Consciousness, on the other hand, does not appear to have these attributes. First, conscious states are not under the control of the will. Although conscious states can be to some extent modulated – by substances like drugs and alcohol, by practice like meditation (Manuello et al., 2016), and by attention itself, which selectively processes information that, thereby, can enters conscious experience –, it simply happens to us to be conscious every morning when we wake up from sleep. In contrast, consciousness itself seems to be a precondition to exert voluntary control of behavior. Second, consciousness appears to be a self-sustaining process (a stream or a flow) that extends through two dimensions, the dimension of the level of vigilance or arousal, and the dimension of contents (Nani et al., 2013; Cavanna and Nani, 2014). Within these two dimensions it is possible to have different degree of consciousness, depending on how much a waking person is vigilant, alert or aroused, and on how vivid and intense the contents of the experience are.

The concepts of attention and consciousness, therefore, can be psychologically separated. The point is now to see whether or not the distinction at the conceptual level can be maintained at the neurophysiological level. Do consciousness and attention have different neural correlates? If so, what is the relationship between these two functions? Can attention be elicited in individuals having unconscious perception? And, conversely, are there cases in which consciousness can occur without attention?

Over the years several experiments have been conducted in order to answer these questions. This paper discusses the conceptual issues that stir the debate about the dissociation of consciousness and attention and reviews the most relevant studies in the neuroscientific literature that have tried to identify the neural correlates at the root of these processes. In light of the results of this research, the final section proposes a model of neural interaction between consciousness and attention capable of reconciling the different positions.

## Separate Functions for Attention and Consciousness

Broadly speaking, there is common agreement on the idea that attention is a brain function capable of selecting relevant information from our sense data. In other words, “the concept of attention refers to one of the basic characteristics of cognition, namely the capacity to voluntary and involuntary give priority to some parts of the information that is available at a given moment” (Naghavi and Nyberg, 2005). As we have already noted, attention has the significant property of being either voluntary or involuntary. This aspect is of fundamental importance, as it allows to distinguish between two types of attentional processes: a top-down attention and a bottom-up attention (Kim et al., 1999; Rosen et al., 1999; Naghavi and Nyberg, 2005). Top–down attention derives from endogenous factors and is characterized by a voluntary control exerted by the conscious mind in order to concentrate on a particular feature (feature-based attention), object (object-based attention) or region in space (focal attention). It is a high-level form of attention capable of selectively exerting concentration on different aspects of a perceptual scene. In turn, bottom-up attention is a low-level form of attention which is elicited by exogenous factors; it is therefore stimulus-driven, automatically triggered by stimuli capable of attracting one person’s focus.

Undoubtedly, consciousness is strictly related to both types of attention. However, there seems to be a curious asymmetry between the two types of attention with regard to their relationship with consciousness. On the one hand, it seems that bottom-up attention can direct consciousness on a certain stimulus. On the other hand, it seems that consciousness can direct top–down attention on a certain stimulus. In other words, it seems that bottom–up attention *precedes* consciousness, whereas top–down attention *follows* consciousness. Evidence for this can be seen in certain types of epileptic crises affecting both the level and contents of consciousness. During complex partial seizures, for instance, patients can show an impairment of the voluntary control of attention (Johanson et al., 2003). In particular, in those patients top–down attention is so much affected as to be described as “forced attention,” because it involves the narrowing of the focus of attention and the absence of the voluntary control of its direction. Nonetheless, although it seems more likely that the dissociation may be possible for bottom–up attention than for top–down attention, experiments and clinical reports show that dissociation is possible for both types of attention.

With regard to top–down attention, a number of experiments show that it is possible to deploy attention to a stimulus that remains unavailable to consciousness (Koch and Tsuchiya, 2007; van Boxtel et al., 2010). This effect has been reported in the attentional manipulation of non-conscious priming and adaptation, as well as in the attentional cueing of invisible stimuli (Ansorge and Neumann, 2005; Kiefer and Brendel, 2006; Sumner et al., 2006). Non-conscious priming is improved by feature-based (Melcher et al., 2005; Tapia et al., 2010), spatial (Kentridge et al., 2008; Finkbeiner and Palermo, 2009; Van den Bussche et al., 2010), and temporal attention (that is, cueing when the prime or targets appear) (Naccache et al., 2002). The force of adaptation to perceptually invisible (features of) stimuli such as orientation (He et al., 1996; Montaser-Kouhsari and Rajimehr, 2004; Kanai et al., 2006; Bahrami et al., 2008a, b; Shin et al., 2009) or the gender of faces (Shin et al., 2009) is enhanced by increasing feature-based and spatial attention to these attributes. Furthermore, there is evidence that attention can be deployed without conscious registration of a stimulus. This has been showed by studies investigating attentional cueing effects on sub-threshold or invisible stimuli (Rajimehr, 2004; Jiang et al., 2006; Sato et al., 2007; Lin et al., 2008, 2009; Meteyard et al., 2008; Tsushima et al., 2008; Bauer et al., 2009). A study showed that the random motion of dot stimuli, whose coherency was so low that subjects cannot distinguish their directions above chance, was more distracting for a concurrently performed central task than the motion of stimuli with high coherency (Tsushima et al., 2006).

With regard to bottom–up attention, the blind vision (Weiskrantz, 1997) and other types of zombie behaviors (Koch and Crick, 2001) show that we can cope with the environment, at least to some extent, without the help of consciousness. For instance, in affective blinsight patients exhibit non-conscious perception of basic emotions (Celeghin et al., 2015), while in somnambulism or sleepwalking a person can move and even drive without conscious perception (Hughes, 2007). This kind of unconscious processing has been also observed in neurological patients experiencing neglect and extinction (Vallar, 1998), limbic status epilepticus (Monaco et al., 2005; Cavanna, 2008), as well as in a number of neuroimaging studies on healthy individuals (Koch, 2006). In particular, the condition of blindsight, in which individuals with lesions of the visual areas are able to avoid obstacles and to point to visual stimuli, provides evidence that both top–down attention and bottom–up attention can occur without consciousness. For example, it has been reported that “the blindsight patient GY has the usual reaction-time advantages for the detection of targets in his blind visual field when attentionally cued, even when the cues are located in his blind field” (Kentridge et al., 2004). There is, therefore, evidence that attention can occur without conscious processing, and that attentional selection can modulate the elaboration of unconscious stimuli (Naccache et al., 2002; Kentridge et al., 2008). Furthermore, it has been reported that attention can be oriented to the location of a target stimulus that remains invisible (McCormick, 1997; Woodman and Luck, 2003). In other words, evidence suggests that simple or single targets do not require attentional selection for conscious processing (Wyart and Tallon-Baudry, 2009).

Experiments show that it is also possible to have a conscious experience of an object or of an attribute of an object without actually paying attention to the object or its attributes (Koch and Tsuchiya, 2007; van Boxtel et al., 2010). As a matter of fact, we are generally conscious of the world that surrounds us without directing explicitly attention to all its elements, that is, without exerting high-level attention on specific aspects of the visual scene. In other words, top–down attention does not need to participate in perceiving the gist of a certain scene (Li et al., 2002; Larson and Loschky, 2009). Other evidence of conscious perception with no direct attention processing comes from studies based on pop-out effect, iconic memory, partial reportability, and dual-task paradigm (Braun and Sagi, 1990; Braun and Julesz, 1998; Block, 2007; Koch and Tsuchiya, 2007; Tsuchiya and Koch, 2008; Lamme, 2010). In particular, it has been observed that the gist of a natural scene as well as the gender and identity of a face picture can be reported under dual-task conditions (Mack and Rock, 1998; Reddy et al., 2004; Reddy et al., 2006; Torralba et al., 2006; Alvarez and Oliva, 2008, 2009).

All the cases described above prove that attention and consciousness can be dissociated and that some aspects of the world can be perceived without consciousness. At least for performing simple actions, which are profoundly based on innate schemas or apprehended automatisms, as well as for paying attention to simple stimuli, consciousness appears to be not essential. This seems counterintuitive, as we are used to think that consciousness is one of the most important properties of our brain, without which we would not be who we are. It is the fact that we are conscious that makes us behave the way we do. For example, we would not be able to learn to speak a new language or to play a musical instrument without being conscious of what we are actually doing. Consciousness, therefore, seems to be fundamental for dealing with unexpected situations and new stimuli, and for performing novel tasks (Baars, 1997). Furthermore, sophisticated emotional experiences cannot be appropriately evaluated by unconscious individuals (Damasio, 1999). Consciousness is also necessary for making decisions, for the voluntary control of actions, for making plans and programs for the future, for recalling memories from the past and for building a sense of self (Baars, 1997). In general, it seems that all our major mental functions, such as reasoning, creative thought, imagination, empathy (Haladjian and Montemayor, 2016), evaluation of complex feelings (Tsuchiya and Adolphs, 2007), memory retrieval, and action planning can occur and develop in the presence of consciousness (Zeman, 2001).

The above considerations suggest that the functional roles of consciousness and attention are radically different. This position is also compatible with an evolutionary framework that considers consciousness and attention two distinct functions and adaptations of biological organisms (Montemayor and Haladjian, 2015). Within this picture, attention is seen as a primitive cognitive function and one of the earliest adaptations of the nervous system, capable of selecting and filtering relevant information for higher-level processing. Types of attention evolved independently and prior to phenomenal consciousness, and allowed the representations of complex multi-feature objects and their maintenance in working memory systems. This was an essential step to develop consciousness, as attention provided the scaffolding for the elaboration of more complex cognitive functions beyond the detection of simple features, including object tracking, visual search tasks, and object recognition (Haladjian and Montemayor, 2015). Therefore, dissociation between the two faculties can also be sustained from an evolutionary perspective.

In sum, consciousness seems to be a process capable of allowing the elaboration of information so as to construct a survey of what is going on inside and outside the body, while attention seems to be the capacity of the mind to shift and appreciate the sensory relevance from one perception to another. In other words, the conscious faculty of the mind can be thought of as a synthesizer, whereas the attentional faculty of the mind can be thought of as an analyzer (van Boxtel et al., 2010). This difference at the conceptual, psychological, and evolutionary level is supposed to be reflected at the neurophysiological level into distinct neural correlates for consciousness and attention.

## Brain Mechanisms at the Root of Consciousness and Attention

### Neural Correlates of Conscious Processing

The neural correlates of consciousness have been defined as the minimal neural mechanisms that are together necessary and sufficient for experiencing any conscious percept (Crick and Koch, 1990). As we have seen, consciousness is a process that unfolds along two dimensions (wakefulness and phenomenal contents). The quest for the neural structures that are important for the level of consciousness has come from classic and modern lesion inquiries, as well as from fMRI investigations, which show that consciousness is supported by a complex interplay of different networks, including the ascending reticular activating system (ARAS) in the brainstem, the non-specific nuclei of the thalamus, and the widespread thalamocortical projections to anterior cingulate, posteromedial cortex and fronto-parietal association cortices (Tsuchiya and Adolphs, 2007; Cavanna et al., 2013).

The level of vigilance can be modulated by the dynamics of resting state and task-engaged networks. According to the ‘default mode’ paradigm of brain function, a system of extensively interconnected cortical regions located mainly on the medial portion of the hemispheres, which is more active during rest than during perceptual and attentional engagement with the environment, is supposed to be crucial to the maintenance of consciousness (Raichle et al., 2001; Nani et al., 2013). In turn, the posteromedial parietal areas (posterior cingulate, retrosplenial cortex and precuneus), together with the medial frontal, anterior cingulate and lateral parietal cortices are more active when the brain is engaged in internal monitoring and in processing information related to self (Cavanna and Trimble, 2006; Cavanna, 2007). This functional network exhibits strong connections not only between its components, but also with fronto-parietal association areas and non-specific thalamic nuclei (Parvizi et al., 2006).

The neuroscientific research on the phenomenal contents of consciousness highlights the activation of structures that are thought to be involved in processing specific conscious percepts. Studies on the neural correlates of phenomenal consciousness have investigated the conditions in which the same sensory information can be processed in presence or in absence of awareness (Moutoussis and Zeki, 2002). This research provides evidence that the initial steps of the conscious perception of a visual stimulus occurs in the very same areas that are activated when the perception of the stimulus is unconscious; what varies between the two conditions is that in the former brain activity is much more intense than in the latter. These studies have led to the identification of important brain nodes, whose activity with a certain degree of intensity within a network is supposed to be fundamental in order to progressively build a conscious perception of the features or objects of the world.

Let us take for example the case of visual consciousness. It has been observed that within the ventral visual system the area V4 performs an elaboration that is essential for having the subjective and phenomenal experience of color (Zeki, 1973, 1983). This area is also supposed to be involved in the selective extraction of features related to shape and depth representation (Roe et al., 2012). It seems therefore that if V4 is selectively damaged (due to a lacunar stroke, for instance), the individual will be unable to experience color. Conversely, if V4 is electrically stimulated (during brain surgery, for instance), the patient will experience color. However, this has been debated (Cowey and Heywood, 1997) and thus far there is no consensus on the matter (Roe et al., 2012). It is more likely that the activity of this region might be essential but not sufficient for the conscious perception of color. According to a number of fMRI studies, in order to have a full-fledged visual conscious experience something further needs to be added, namely, a complex and dynamical interaction among other brain areas, especially those, it has been claimed, of the fronto-parietal network (Dehaene and Naccache, 2001; Naghavi and Nyberg, 2005).

The suggestion that consciousness depends on the global activation of a large-scale cortical network has led to the idea that the neural correlates of visual conscious perception are not to be found in the primary or secondary visual cortices, but, rather, in the association activity of the fronto-parietal system (Dehaene and Naccache, 2001; Rees et al., 2002; Bor and Seth, 2012). Similarly, it has been proposed that the neural correlates of visual consciousness should be divided into primary and secondary brain areas, whose early activity in the occipital lobe may support the first perceptual discriminations among stimuli and later activity in fronto-parietal areas may support the integration of different visual features that are contingent on the outcomes of the earlier perceptual processing (Pins and Ffytche, 2003). Reentrant signaling mechanisms are supposed to lie at the root of these different types of elaboration (Seth and Baars, 2005). This recursive processing is considered to be the combined flow and integrated outcome of afferent and recurrent activity across a series of cortical areas (Pollen, 2003). It therefore appears to be one of the predominant forms of communication between brain networks (Di Lollo et al., 2000).

Important reentrant circuits are not only cortico-cortical but also thalamocortical. Especially with regard to the thalamus, it has been proposed that this complex structure may play a pivotal role in supporting consciousness (Ward, 2011). The numerous nuclei of the thalamus are extensively connected with the cortex, from which they receive feedback projections (Nieuwenhuys et al., 2007). It has been hypothesized that especially the thalamic reticular nucleus (TRN) may play a role in modulating consciousness by regulating the local 40-Hz oscillations that are observed in many parts of the brain (Newman, 1995; Min, 2010). Indeed, the capacity of the thalamus to synchronize cortical activity has been repeatedly observed (Llinas et al., 1998, 1999; Herrero et al., 2002). Furthermore, it has been observed that the abolishment of inhibitory interactions between the neurons of the TRN and other thalamic neurons significantly increases absence epileptic seizure-like and low-frequency synchronous oscillations within the dorsal thalamic nuclei (Huntsman et al., 1999). This finding provides evidence that such inhibiting mechanism might play a role in preventing the neural hyper-synchrony that characterizes generalized seizures and their accompanying state of unconsciousness (Steriade, 2005). Notably, only two places in the brain can abolish consciousness if damaged bilaterally: the ARAS and the intralaminar nuclei of the thalamus. These thalamic nuclei are intensively connected with both the ARAS and much of the rest of the brain (Ward, 2011). These diffusely projecting thalamic neurons are supposed to constitute a pathway capable of subserving the dimension of the level of consciousness by propagating synchronous oscillations across the brain so as to create a coherent baseline of neural activity (Jones, 2001, 2002, 2009). Within this picture, the thalamic nuclei, in combination with the brainstem arousal system, can determine and maintain thalamo-cortical synchronization at 40 Hz (vigilance or wakefulness) or at much lower frequencies, in the delta (2–3 Hz) range (sleep) (Ward, 2011).

The relationship between consciousness and synchronous neural activity has been repeatedly emphasized (Tononi and Edelman, 1998a, b; Rodriguez et al., 1999; Srinivasan et al., 1999; Edelman and Tononi, 2000; Engel and Singer, 2001; Ward, 2002, 2003; Melloni et al., 2007). In particular, the process of brain synchronization appears therefore to be a fundamental ingredient for binding together a multitude of attributes within a single conscious experience (Singer, 1999).

Undoubtedly, this complex picture makes consciousness a matter of degrees. A phenomenal content of consciousness is progressively constructed and refined passing through the elaboration of different brain areas until it is completely processed. It is as if the primary and secondary sites of perception do an early and preconscious draft of the content; then this ‘protocontent’ is passed on for further processing to other areas, which are in a higher position in the cortical hierarchy, until the content reaches the final stage and is broadcast throughout the global workspace of the fronto-parietal system, where it becomes eventually conscious. Three stages have been proposed to account for this elaboration: subliminal, preconscious, and conscious (Dehaene et al., 2006). The first stage (i.e., subliminal) is not strong enough to produce the emergence of conscious experience. The second stage (i.e., preconscious) is strong enough but, without the help of attention, cannot produce a content that enters the global workspace. In other words, the preconscious stage is supposed to be confined to sensori-motor processors within occipito-temporal loops and its contents, though they can cause priming at multiple levels, cannot be reported. The third stage (i.e., conscious) is strong enough and, at the same time, can produce a reportable content that enters the global workspace when it is processed under the light of attention. In this theoretical model, the movie of consciousness is directed by attention, which decides which content can or cannot play its part on the theater of conscious experience.

**FIGURE 2:**
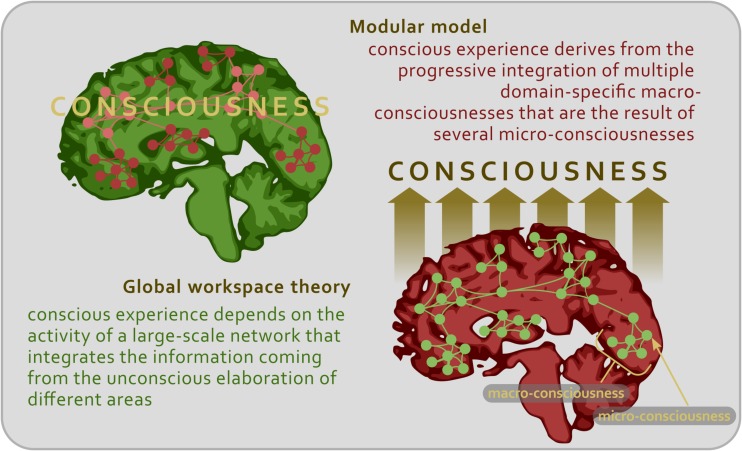
Schematic representation illustrating the two main theoretical approaches to the study of consciousness.

It can be claimed that this elaboration may apply to every type of conscious content, not just to the visual ones. Each phenomenal content (be it visual, auditory, tactile, gustatory, and somatosensory) needs to be analyzed in increasingly sophisticated steps so as to emerge consciously as an object of thought and perception. As we have seen, along this chain of processing phases, certain brain nodes might be more fundamental than others (such as V4 for color representation), the impairment of which may lead to the lack of capacity to experience the relative conscious feature (for instance, achromatopsia in case of V4 disruption or lesion). At this point, the absence or defects of the conscious percept could be interpreted in two different ways. On the one hand, it could be argued that if the final stage of conscious processing is not accomplished, that is, if a certain aspect of the phenomenal content does not enter the fronto-parietal system, then it is not possible to consciously perceive that aspect. On the other hand, it could be argued that the aspect of the content, which is not consciously perceived, remains under the threshold of consciousness only because the function of the disrupted node cannot be replaced by the compensation of other areas (Figure 2).

This second view applies the idea of modularity not only to simple mental functions, such as sensory detection, but also to higher-order mental functions, such as memory formation, language and consciousness (Sperber, 2001; Barrett and Kurzban, 2006; Carruthers, 2006), and contends that the so-called ‘final stage’ in which the phenomenal content is processed by the fronto-parietal system is *the* necessary and sufficient stage of consciousness (Nani and Cavanna, 2012). In contrast, each brain node would be already in itself both necessary and sufficient for the emergence of a certain feature in the conscious experience. In other words, the neural structures responsible for the contents of consciousness may rely on the activity of domain-specific modules capable of processing in parallel different chunks of phenomenal experience. Neurological and neuropsychological conditions provide evidence that consciousness can be specifically impaired (Nani and Cavanna, 2012). For instance, patients with epilepsy (especially during focal seizures) can show the selective disruption and preservation of cognitive performances, behavioral responses, and conscious phenomena (Gloor, 1986, 1990; Porter, 1991). Studies on split-brain patients highlighted subtle cognitive reorganizations in which specific contents of consciousness are confined to one hemisphere (Gazzaniga et al., 1963; Sperry, 1966; Teng and Sperry, 1974). In turn, neuropsychological conditions, such as blindsight, anosognosia, prosopagnosia, and neglect, show that certain features of conscious experience can be selectively damaged or abolished (Tranel and Damasio, 1985; Berti and Rizzolatti, 1992; Bisiach, 1992; Weiskrantz, 1997). Thus, rather than being represented in a single brain central system or global workspace, a content of experience may become conscious in the very neural structure that analyzes its attributes (Kanwisher, 2001). In a sense, the primary cortex would produce a set of micro-consciousnesses specific for each sensory modality; in turn, these micro-consciousnesses would be assembled by the secondary cortical areas in a macro-consciousness, always specific for each sensory modality, and, eventually, the diverse macro-consciousnesses would be combined in a full-blown conscious scenery (Zeki, 2007) (Figure 2).

The two approaches to the neural correlates of consciousness have both advantages and disadvantages. As we have seen, the model based on modularity seems to be more supported by clinical evidence, as there are many conditions in which consciousness appears to be variously fragmented and impaired, in such a selective way which it makes convincing the idea that the brain structures underlying consciousness may be based on a modular architecture (Nani and Cavanna, 2012; Gazzaniga, 2018). An exaggerated modular stance, however, would lead to an unjustified proliferation of minute pieces of consciousnesses. Since any brain processing area should in principle produce a micro-consciousness, then we should expect to find as many micro-consciousnesses as there are brain processing areas. Still, some brain sites, such as the cerebellum and the basal ganglia, do not appear to be directly involved in building a conscious experience and, consequently, do not produce micro-consciousnesses *per se*. Cerebellar agenesis is a rare condition which is mainly associated with motor impairment, postural and balance disturbances (Glickstein, 1994; Velioglu et al., 1998). The absence of cerebellum or its functional disruption can lead to deficits that are not only related to motor processing but also to cognitive functions (Fiez et al., 1992; Arrigoni et al., 2015; Dahlem et al., 2016). Similarly, lesions to basal ganglia are associated with motor deficits and with various cognitive processes (linguistic, attentional, mnestic, and executive); in particular, their dysfunction has been related to neurodevelopmental disorders (Riva et al., 2018). Therefore, both cerebellum and basal ganglia seem to be fundamental for a normal neurocognitive development (Stoodley, 2016) and for the unconscious elaboration of certain cognitive features, but to date there is no evidence that they are directly involved in phenomenal conscious experience.

The modularity model runs into difficulties when it is required to explain why the activity of certain assemblies of neurons correlates well with conscious experience, while that of others does not (Cavanna and Nani, 2014). What is more, at one point all the different macro-consciousnesses eventually need to converge and be combined in a global experience. So, the unified conscious scenery might be dependent on the activity of large-scale association networks, even if the various minute aspects of the phenomenal contents are not.

In turn, the central model based on the activity of a large-scale fronto-parietal system seems to be capable of accounting well for access consciousness (that is, the availability of representational contents for use by different cognitive systems; Block, 1995) but not for the synthesis of different percepts that one would expects from a unitary and global scene of phenomenal consciousness. Top–down attentional amplification is supposed to be a necessary requirement for the conscious experience of contents, as it can mobilize and make available to the global workspace the contents produced by domain-specific processes (Dehaene et al., 1998). It seems, therefore, that the fronto-parietal system is constrained by attention to work serially rather than in parallel. This would create a bottleneck that impedes the binding of different features of the phenomenal world. Furthermore, the view that conscious processing is solely supported by one system in the brain has been criticized (Nani and Cavanna, 2012; Gazzaniga, 2018). First, the boundaries of the fronto-parietal system are vague and it is not exactly clear which frontal and parietal areas are effectively parts of the system and which are not. Secondly, the exact role of the frontal components in the fronto-parietal system is not as yet understood (Boly et al., 2017). There are many clinical cases in which lesions of frontal areas have been observed without impairment of consciousness. For instance, after brain surgery that involved the bilateral resection of prefrontal cortical areas, patients were observed to be fully conscious (Penifield and Jasper, 1954; Kozuch, 2014; Tononi et al., 2016). In one case, a woman exhibited a massive bilateral prefrontal lesion, which involved the right basal, superior, medial and lateral prefrontal cortex, and the left medial orbitofrontal, frontopolar, and frontal gyri; however, even though she had evident deficits in cognitive functions, her consciousness and perceptual capacities were not affected (Markowitsch and Kessler, 2000). It is well known that lesions involving medial prefrontal regions, in particular those affecting the anterior cingulate cortex, can produce akinetic mutism, a condition in which patients still have the capacity to visually track stimuli but are unable to respond to commands (Cairns et al., 1941). Typically, individuals that recover from this condition report that they lacked any motivation to respond to stimuli, even though they were fully conscious of them (Damasio and Van Hoesen, 1983). Furthermore, when Broca’s area is injured, speech production is impaired, but the damage does not produce a relative loss in conscious speech perception (Blumenfeld, 2010). Interestingly, damage to frontal areas can slightly increase the threshold for detecting brief (16 ms) and masked visual stimuli, but patients are nonetheless able to perceive them (Del Cul et al., 2009), which suggests that frontal areas might have the role of modulating consciousness rather than directly contributing to it (Kozuch, 2014).

Other authors, however, argue against this view (Odegaard et al., 2017). In particular, evidence that the frontal areas (especially the prefrontal ones) might be involved in generating consciousness comes mostly from experiments on conscious vision. A number of studies have highlighted that transcranial magnetic stimulation and lesion to human prefrontal cortex can produce impairment in many aspects of visual perception (Turatto et al., 2004; Ruff et al., 2006; Philiastides et al., 2011; Lee and D’Esposito, 2012; Ritzinger et al., 2012; Chiang et al., 2014; Rahnev et al., 2016). And some neuropsychological studies have shown that patients with unilateral lesion to the prefrontal cortex frequently exhibit deficits in visual tasks (Barcelo and Knight, 2002). Still, it should be observed that the deficits reported in these clinical and experimental cases largely involve the modulation of visual experience rather than the contents *per se*. In other words, prefrontal areas seem to be responsible for regulating the ability to detect visual targets by modulating activity in extrastriate regions and temporoparietal cortices (Voytek et al., 2010). Therefore, the main role of the prefrontal components of the fronto-parietal system seems to add aspects of cognitive relevance to contents that appear to be already formed, so that a damage or lesion to these areas may cause disturbance to conscious perception caused by an impairment of executive functions and attention, but not the loss of specific contents of subjective experience.

There is, in contrast, abundant clinical evidence that damage to posterior brain areas can selectively disrupt consciousness. For instance, lesions of the right fusiform face area produce prosopagnosia, a condition in which faces cannot be recognized (Barton and Cherkasova, 2003). We have already seen that lesions to the inferolateral occipital cortex can cause achromatopsia (Barton, 2011), which in severe cases is accompanied by unawareness of the deficit (von Arx et al., 2010). In turn, damage to the occipital cortex can cause selective blindness as well as visual agnosia, an incapacity of identifying objects, or simultanagnosia, an incapacity of perceiving more than one object at a time (Farah, 2004). Loss of somatosensory percepts, instead, are caused by lesions in postrolandic cortex, while impairment in the comprehension of speech and prosody are caused by damage to left and right angular gyri (George et al., 1996). Moreover, loss of motor awareness can be produced by lesions of the inferior parietal lobule (Sirigu et al., 2004), and deficits in the perception of single words or whole phrases can be produced by lesions of the left lateral temporal cortex (Blumenfeld, 2010). So, clinical evidence strongly suggests that temporal, parietal, and occipital areas may be considered as ‘posterior hot zones’ capable of playing a direct role in the construction and specification of the contents of phenomenal consciousness (Boly et al., 2017).

Important questions, however, remain as yet unanswered. For instance, how does the binding between different phenomenal contents occur? How can the brain weave all the diverse percepts in a unitary conscious scene? It should be noted that both the modular model and the global workspace model do not offer a solution to this problem. With regard to the former, the phenomenal contents of conscious experience seem to be processed independently of each other; with regard to the latter, a central system must process information slowly and serially and, as a consequence, work under the constraint of attending to each content one at a time. But this is not what we daily experience. In fact, we are always conscious of a variety of inputs coming from the senses. The common idea that consciousness is a serial processing of information cannot be correct. This is undoubtedly true for attention, but not for consciousness, as phenomenal contents need to be elaborated in parallel if they have to be parts of a unitary experience. This is another distinction between these two brain faculties. Furthermore, we are always conscious of a perceptual global scene within which different sensory features seem to occur *simultaneously*. Therefore, the binding performed by the brain does not only require the integration of various perceptual elements, but also the temporal merge of these elements. And this is another issue that needs an explanation. Not only we are able to consciously perceive different aspects of reality together, but we perceive them in a common temporal framework, even though it is known that sensory perceptions are processed at different timescales. For instance, visual information reaches awareness in about 60 ms, while auditory information in about 15 ms (Celesia, 1976; Lesevre, 1982; Kopinska and Harris, 2004). There is, therefore, a temporal gap between the two sensory modalities, but, quite astonishingly, when constructing conscious experience of multisensory stimuli, the brain is able to shift the perceived time of the visual component toward that of the auditory component (Lewald and Guski, 2003). The time of occurrence can be adjusted both when a visual stimulus is presented before an auditory stimulus and when the auditory stimulus is presented before a visual stimulus (Jaekl and Harris, 2007). This temporal adjustment is crucial for perceptual coherence and the mechanisms at its root are fundamental for understanding consciousness. Sooner or later, the neuroscience of consciousness will have to address these important computational issues. This is not the place to propose a comprehensive theory of consciousness; however, in section ‘The Construction of the Conscious Experience’ we try to give some suggestions as to how to tackle the fascinating intricacies of the binding problem.

### Neural Correlates of Attention Processing

Over the last years, a number of fMRI and PET studies have tried to shed light on the nature of the neural mechanisms that underlie attention, which is defined as the capacity to voluntarily or involuntarily give priority to some parts of the information that is available at a given moment (Naghavi and Nyberg, 2005). Investigations have revealed a distributed system of brain areas that control attention by enhancing and regulating the elaboration of specific aspects of information, and have shown that attention correlates mostly with activation patterns in the bilateral parietal and dorsolateral prefrontal cortices (Pessoa et al., 2003). In particular, areas in the frontal eye field, superior parietal lobule and intraparietal sulcus have consistently been found to be active in various tasks involving spatially directed attention (Gitelman et al., 1999; Kim et al., 1999; Rosen et al., 1999; Hopfinger et al., 2000; Beauchamp et al., 2001; Corbetta et al., 2002). Other activations frequently observed involve the middle and inferior frontal gyrus, inferior parietal lobule, and anterior cingulate cortex (Naghavi and Nyberg, 2005). Activity in the parietal and frontal areas has been reported not only for visual attention tasks, but also in attentive tasks involving other sensory modalities. For example, a PET study investigated brain activations while participants were attending to spectral and spatial features of sounds (Zatorre et al., 1999). In addition to the bilateral activation of auditory cortex, authors reported increases in the activity of right superior parietal, right dorsolateral frontal, and right premotor regions. Another PET study provided further evidence for these findings: when listeners were engaged in auditory spatial attention tasks, a set of frontal, temporal and parietal regions was typically activated (Lipschutz et al., 2002).

**FIGURE 3:**
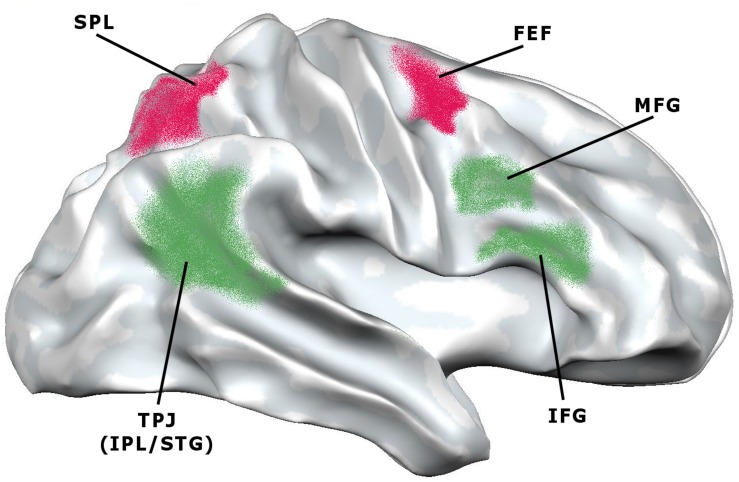
Lateral view of the brain showing the areas involved in the dorsal attention network (DAN) and ventral attention network (VAN) (adapted from Corbetta and Shulman, 2002). DAN (red): FEF, frontal eye field; SPL, superior parietal lobule. VAN (green): IFG, inferior frontal gyrus; IPL, inferior parietal lobule; MFG, middle frontal gyrus; STG, superior temporal gyrus; TPJ, temporo-parietal junction.

These results suggest the existence of a multimodal large-scale attentional system capable of operating independently of the nature of performance (be it visual, auditory, etc.), along with additional brain areas that may be recruited according to current task demands. Within this multimodal system, two main attentional networks have been identified (Corbetta and Shulman, 2002). One follows a dorsal pathway and connects the superior parietal lobule, the intraparietal sulcus and the frontal eye field; the other follows a ventral pathway and connects the temporoparietal junction (which is at the intersection of the inferior parietal lobule and the superior temporal gyrus) and the middle and inferior frontal gyri. The former is known as the dorsal attention network (DAN) and is mainly associated with goal-directed stimulus-response selection. The latter is known as the ventral attention network (VAN) and is mainly associated with the detection of behaviorally relevant stimuli. In other words, the DAN is supposed to regulate the top–down voluntary deployment of attention to locations or features, while the VAN is supposed to mediate shifts of attention when triggered by unattended or unexpected stimuli (Figure 3). This may lead to think that DAN supports top-down attention, while VAN supports bottom-up attention. Still, as we are going to see, the conceptual distinction between the two types of attention has been questioned at the neural level, as it could not correspond to distinct patterns of networks’ activations.

The two networks seem to be slightly asymmetric in their functional profiles. Neuroimaging data show that the DAN has a bilateral functional organization, whereas the VAN might be more lateralized to the right hemisphere (Corbetta et al., 2008). However, these results have been questioned by other studies, which have found bilateral activations of the VAN or activations in the left temporoparietal junction during attentional tasks (Downar et al., 2000; Weidner et al., 2009; DiQuattro and Geng, 2011). Different functional activations between left and right temporoparietal junctions were reported in a location-cueing paradigm (i.e., Posner task): the left temporoparietal junction was more activated in responses to both invalidly and validly cued targets, whereas the right temporoparietal junction exhibited stronger activations in responses to invalidly rather than validly cued targets (Doricchi et al., 2010). Interestingly, in the two hemispheres the temporoparietal junctions present different patterns of structural connectivity. The right temporoparietal junction exhibits higher connectivity with the insula, while the left temporoparietal junction exhibits higher connectivity with the inferior frontal gyrus (Kucyi et al., 2012). Asymmetries are probably due to functional different patterns in the activations of the right and left VANs, which, in turn, may derive from different routes of anatomical connections. However, it is important to highlight that DAN and VAN are supposed to work in close synergy, even though they are well recognizable and anatomically segregated cortical networks, in which certain nodes have functional specializations for attentional control (Vossel et al., 2014).

The two categorizations of attention (i.e., top–down and bottom–up) can be well defined with regard to their origin of information, but this conceptual dichotomy may not be perfectly mirrored by distinct patterns of brain activation (Katsuki and Constantinidis, 2014). These authors claim that, although both types of attention can be related to different neural functions and needs, they nonetheless rely upon the same DAN and VAN pathways, so much so that they can simultaneously influence and integrate other processes, such as visual and spatial search. The same set of brain areas within the fronto-parietal network seems to support both top–down and bottom–up attention, by providing a priority map for selection of stimuli on the basis of different source factors. Therefore, authors conclude that, at the neurophysiological level, the distinction between the two types of attention appears to be more arbitrary than real. However that may be, we have seen that both top–down attention and bottom–up attention can be dissociated from consciousness, and that, taken together, imaging studies provide strong evidence that a distributed fronto-parietal system is generally involved in attentional tasks and may function to generate signals (independently of their top–down or bottom–up sources) capable of modulating activity in lower-level sensory and association structures, which further suggests that prefrontal areas might affect conscious experience indirectly, by engaging, disengaging and redirecting attention, in accordance to the contingencies of the current situation.

## Discussion

### The Interplay of Conscious and Attention Processing

Both psychological and neurophysiological considerations make possible to clearly distinguish between consciousness and attention. Although they are separate brain processes with different functions, consciousness and attention are strictly intertwined. While attention may be not essential for the construction of the conscious phenomenal contents *per se*, it is nonetheless fundamental for their conscious access. For instance, visual recognition depends on attention even for highly familiar and meaningful materials (Rees et al., 1999). This suggests that distributed interactions between modality-specific posterior areas and fronto-parietal regions are likely to subserve both attention and focal awareness (Rees and Lavie, 2001).

The studies reviewed in this paper provide evidence that attention and phenomenal consciousness involve overlapping patterns of activity in temporal and parietal cortices. With regard to phenomenal consciousness, these patterns of activations have been associated with the integration of distributed representations in multiple brain sites, which, rather than converging to a single fronto-parietal system, seem to be mainly localized in temporal, parietal, and occipital areas (Boly et al., 2017). Therefore, the global neuronal workspace, in which the phenomenal contents of experience emerge, might be formed by the dynamics of different associative networks in the back of the brain; these higher-order networks receive information from sensory domain-specific areas or modules, whose activity is the first cortical step toward the formation of a full-blown phenomenal content. In turn, the temporo-parietal-occipital workspace would be continuously scanned and accessed attentively by DAN and VAN. These two attention networks can be considered as a mental faculty representing the information that is dominant at each moment within the brain.

According to this view, conscious experience would emerge with the contribution of different processes, following a precise hierarchical pathway. First, the brainstem and the thalamocortical projections are required to maintain the state of vigilance or alertness. This function is of fundamental importance, as it enables all the other parts of the brain which contribute to consciousness to operate at the same baseline. We hypothesize that the maintenance of wakefulness may be essential for binding different aspects of reality. It is the fact that brain areas can process information at the same baseline of vigilance that makes them capable of binding the various features of this information. Sensory primary cortices begin the construction of a specific phenomenal content through different layers of elaboration. Then this initial processing of what we can consider a ‘protocontent’ is distributed within the associative networks in temporal, parietal and occipital cortices, where it can become a full-blown conscious content. At the root of these neural mechanisms there is a continuous mutual exchange of information between specialized, associative and integrative areas. This recurrent processing, which has been proposed to be a key step in visual consciousness (Lamme, 2003, 2004), seems to be essential for building any other type of conscious experience, as it enables a widespread diffusion of information between brain regions processing different attributes of the conscious scene. However, to maintain the coherence as well as the unitary nature of the conscious scenery, we propose that this recurrent processing may develop into a temporal alignment of different percepts. Perceptual binding might therefore be dependent on two neural processes: on the one hand, the same activation baseline supported by brainstem and thalamocortical projections and, on the other, the temporal adjustment of information recurrence. The temporal adjustment is thought to be an intrinsic property of brain networks, which are able to autonomously synchronize with each other by using as benchmark the thalamocortical activation baseline. Changes in short-term synaptic plasticity are supposed to be at the basis of this dynamics that can transform brain networks in time keepers (Buonomano and Maass, 2009). According to this view, every cerebral area has the capacity to process and estimate time. Overall, this mechanism would allow the construction of the global conscious experience, as different phenomenal contents of consciousness can be produced by synchronized neural networks that work in parallel.

### The Construction of the Conscious Experience

As we have seen, synchronization is supposed to be a fundamental ingredient for the construction of conscious experience. Now the idea that at the basis of functional processing there be a specific synchronization between different networks is increasingly gaining interest in the field of connectivity analyses (Lombardi et al., 2017). The dynamic interaction between brain networks has been found to happen at different timescales (Chang and Glover, 2010; Smith et al., 2012; Zhang et al., 2013; Keilholz, 2014), so that it has been proposed that the functional operations of the human brain are scale-free (Eguiluz et al., 2005; He et al., 2010; Zhigalov et al., 2017). In virtue of the coherence among physiological signals with different frequencies, each temporal resolution may provide a different picture of the same phenomenon. Furthermore, each functional network has been found to be characterized by a specific electrophysiological signature involving the combination of different brain rhythms (Mantini et al., 2007). Synchronization (or the coalescence of several neurophysiological rhythms) between large-scale networks might therefore be an essential feature of the human brain functional organization, whose temporal disruption may lead to specific dysfunctions associated with brain disorders (Uhlhaas and Singer, 2012). According to this approach, brain networks are in an endogenous state of dynamical criticality, which is characterized by a greater than random probability of both long-standing intervals of phase-locking and occurrence of great fast changes in the state of global synchrony (Kitzbichler et al., 2009). Network hubs may play a pivotal role in regulating and modulating the dynamics of the synchronization (Vlasov and Bifone, 2017). In particular, the hubs defined as “connector hubs” (i.e., hubs that can connect different networks in virtue of their projections diversely distributed across communities) might tune the connectivity of their neighbors to allow for appropriate integration of information across different neural assemblies (Bertolero et al., 2018).

**FIGURE 4:**
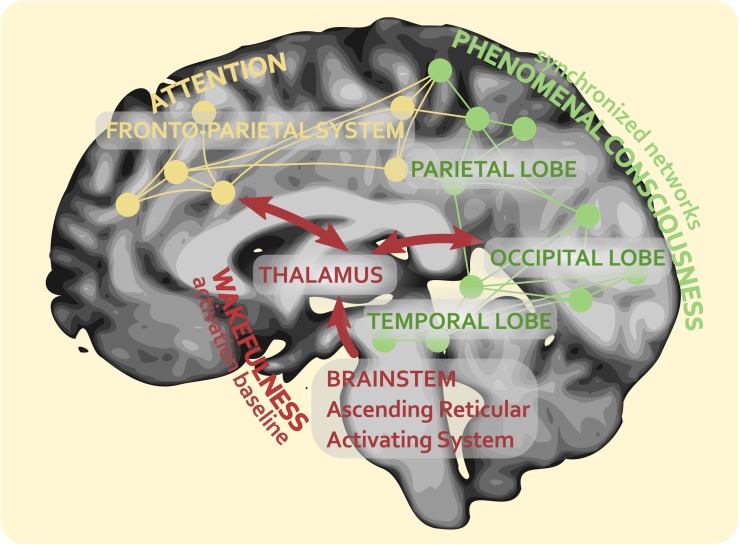
The networks’ synchronization theory (NetSync) and representation of the intersection (and partial overlapping) of the neural correlates of consciousness and attention.

In our theoretical framework, up until the stage of synchronization, phenomenal consciousness is supposed to develop without attention. Attention processing, which, as we have seen, follows an independent route, would enter this picture only afterward, when the fronto-parietal system formed by DAN and VAN begins to focus the attentional resources on specific contents of consciousness, giving to them different degrees of awareness with the regulation of their serial access and availability. Figure 4 outlines this theoretical view. It shows that, from the neural perspective, attention and the two main dimensions of consciousness (wakefulness and phenomenal contents) can be defined as essentially separate but sister processes of the brain, with partially overlapping neural correlates within parietal association networks.

Our proposal can be seen as a third approach to the study of consciousness, which we propose to call the theory of *networks’ synchronization* (NetSync). The NetSync theory of consciousness tries to avoid the disadvantages of the central and modularity models. First, it allows a parallel elaboration of different contents of conscious experience, to which it can attribute different degrees of awareness according to the current focus of attention. Secondly, it puts forward a solution for the binding problem, suggesting that the binding of different phenomenal features may depend on two neural processes: an activation baseline that is sustained by brainstem and thalamocortical projections and a mechanism of temporal alignment that is intrinsic to brain networks which process specific contents of consciousness. Thirdly, it avoids the unjustified proliferation of micro-consciousnesses, as it claims that only the networks that participate in the synchronization can contribute to the global conscious experience. Fourthly, it can account for the dissociation between consciousness and attention, suggesting that it occurs whenever there is a mismatch in the synchronization between the networks underlying the two faculties. In other words, with regard to this last point, it seems that, according to the NetSync model, if there is not a synchronization, because it is briefly delayed or prevented by an experimental paradigm, between the fronto-parietal system of attention and the temporo-parietal-occipital networks of the contents of consciousness, then the stimulus cannot be phenomenally perceived.

Although it is possible, from a logical point of view, to pay attention to something without any associated emotional experience, emotional states and phenomenal consciousness are known to influence the focus of attention as well as to modulate the degree of responsiveness to external stimuli. However, to include in this paper a discussion of the emotional system and its relationship with consciousness and attention would have largely exceeded the boundaries of the review. In particular, we decided to not include in the discussion the role played by the insular cortex and the salience network in modulating consciousness and attention. The salience network, which includes the anterior insula and dorsal anterior cingulate cortex, is important for detection and mapping of external salient inputs and task control (Dosenbach et al., 2007; Seeley et al., 2007; Menon and Uddin, 2010; Uddin, 2015). It is supposed to work in synergy with the DAN and to occupy the apex of the hierarchical organization formed with DAN and the default mode network (Zhou et al., 2018). In turn, the insular cortex has been associated with the emotional modulation of conscious experience (Craig, 2010; Seth et al., 2011). Still, it is debated whether the insula can contribute by processing an essential ingredient of consciousness or just an attribution, albeit important, of a particular emotional and salient flavor to the contents of experience. This last eventuality is supported by the evidence that consciousness is preserved, as well as the capacity of having feelings, after bilateral lesions of the insula (Damasio et al., 2013). So, rather than being involved in phenomenal consciousness (save for the gustatory cortex), the insula might be fundamental for creating self-awareness (Modinos et al., 2009; Manuello et al., 2018). Despite the doubts about the exact role played by the insular cortex in conscious experience, the issue as to whether or not emotional valence might be a necessary varnish to the contents of consciousness and, more generally, to the contents of thought, is an open and intriguing question for future research.

## Conclusion

The conceptual and psychological distinction between consciousness and attention is also reflected at the neurophysiological level. Consciousness and attention are distinct and separate, albeit strictly intertwined, processes going on in the brain. They have different functions as well as different neural correlates. Consciousness has the function of creating a continuous and coherent picture of reality, while attention has the function of attributing relevance to the objects of thought. Consciousness develops along two dimensions, that of wakefulness and that of contents. It can also be conceptually distinguished between phenomenal consciousness (how the world appears to us) and access consciousness (when contents are more or less vivid, intense, and available for focal awareness).

The neural correlates of consciousness are generated by the synchronous activity of sensory and associative networks in the temporal, occipital, and parietal cortices, which elaborate information of specific features of conscious experience. We propose that all these features are bound together in virtue of the temporal alignment of multiple networks, which uses as benchmark the thalamocortical activation baseline. The neural correlates of attention are generated by a fronto-parietal system, within which two main networks can be recognized, the DAN on the one hand, and the VAN on the other. Both neural correlates partially overlap each other in the parietal association networks. In case of mismatch in the synchronization between the networks subserving consciousness and attention, dissociation between these two processes can occur.

Still, even though distinct and separate, each brain process serves the other. Thanks to the role of attention, that incessantly patrols the vast landscape of mental contents, phenomenal consciousness can acquire cognitive relevance. Thanks to the role of consciousness, that creates a consistent scenery of the world, attention can provide focal awareness and make richer our experience. Both are vital brain faculties, and their entanglement is at the core of what it is like to be a human being.

## Author Contributions

AN conceived the idea, drafted and revised the manuscript. JM worked on illustrations and revised the manuscript. LM worked on illustrations and revised the manuscript. DL worked on bibliography and revised the manuscript. TC and FC revised the manuscript.

## Conflict of Interest

The authors declare that the research was conducted in the absence of any commercial or financial relationships that could be construed as a potential conflict of interest.
